# Balloon-assisted retrograde cerebral venography for enhanced visualization of the posterior and lateral dural venous sinuses: a feasibility study

**DOI:** 10.1007/s00234-026-04110-9

**Published:** 2026-07-10

**Authors:** Warda Ahmed, Om H. Gandhi, Sami Almasri, Suehyb G. Alkhatib, Colbey Freeman, Suyash Mohan, Linda J. Bagley, Arti Singh, Nathan Yu, Luis Octavio Tierradentro-Garcia, Ajay K. Wakhloo, Omar A. Choudhri

**Affiliations:** 1 Medical College, Aga Khan University, Karachi, Pakistan; 2 Department of Neurosurgery, University of Pennsylvania, Philadelphia, USA; 3 Department of Radiology, University of Pennsylvania, Philadelphia, USA; 4 Department of Radiology, Tufts University, Medford, USA

**Keywords:** Retrograde cerebral venography, Balloon-assisted venography, Venous sinus stenosis, Venous sinus stenting, Idiopathic intracranial hypertension, Dural venous sinuses

## Abstract

**Purpose:**

The venous phase of DSA offers inferior spatial resolution of cerebral venous anatomy due to contrast dilution. Likewise, retrograde cerebral venography (RCV) suffers from poor contrast retention due to contrast injection opposing the physiological direction of venous drainage. Therefore, this study aims to investigate the feasibility of balloon-assisted occlusion in improving visualization of cerebral venous anatomy during RCV.

**Methods:**

17 consecutive patients who underwent balloon-assisted RCV between September 2023-June 2025 were included. A 9 F balloon guide catheter was positioned in the internal jugular vein or sigmoid sinus. Venography was performed before and after balloon inflation. Using standardized criteria, three neuroradiologists independently assessed visualization quality of venous structures, anatomical furthest extent of contrast enhancement, and pathology delineation. McNemar’s test was used for paired statistical comparisons between visualization with and without balloon occlusion.

**Results:**

Balloon inflation improved rates of “well visualized” pathology from 15.7% to 88.2% (*p* < 0.001, OR = 29.04). The most distant structure visualized shifted from the ipsilateral sigmoid sinus without balloon occlusion (visible in 45.1% of cases) to the contralateral sigmoid sinus with balloon occlusion (visible in 72.5% of cases, *p* < 0.001). With balloon occlusion, homogenous opacification and clear margin delineation of both transverse and sigmoid sinuses showed significant improvements (*p* < 0.001 for both). No procedure-related complications were observed in this limited cohort.

**Conclusion:**

In this feasibility experience, balloon-assisted occlusion during RCV improved visualization of the posterior and lateral dural venous sinuses and associated pathology. Future well-powered studies are needed to validate our findings and determine the definitive safety of this technique.

**Supplementary Information:**

The online version contains supplementary material available at https://doi.org/10.1007/s00234-026-04110-9.

## Introduction

The use of venous sinus stenting for idiopathic intracranial hypertension increased by 80% from 2016 to 2020, reflecting its growing adoption in U.S. neurointerventional practice [1]. However, accurate visualization of cerebral venous anatomy remains challenging. While digital subtraction angiography (DSA) provides excellent arterial delineation, venous structures are visualized only indirectly during the delayed venous phase as contrast passively drains from the arterial circulation, with progressive dilution compromising image quality [2, 3]. These limitations are particularly problematic for visualizing skull base venous anatomy, where the sigmoid sinus and its junction with the internal jugular vein (IJV) exhibit high anatomic variability and are further obscured by adjacent temporal bone structures [4–6].

Early comparative studies demonstrated that direct venography (direct contrast injection into venous structures) provided superior visualization compared to the venous phase of carotid arteriography for evaluating skull base and IJV pathologies, leading to its increased use in clinical practice [4, 7]. Catheter-directed venography has recently been used to assess IJV pseudo-occlusion, where invasive imaging differentiated true occlusions from extrinsic compression that noninvasive computed tomography venography (CTV) had erroneously suggested as complete occlusion in all patients [8]. These studies, describing antegrade venography techniques, typically require navigating past the pathology to position the catheter upstream (e.g., in the superior sagittal sinus), with the proximal catheter potentially obscuring the target skull base anatomy [9].

Among direct venography approaches, retrograde cerebral venography (RCV) via the IJV was first introduced by Gejrot and Laurén in 1964, offering particular advantages for assessment of the dural venous sinuses [10]. The technique provides access to distal venous structures through retrograde IJV catheterization, enabling visualization of the sigmoid sinus, transverse sinus, and associated venous anatomy [10]. RCV has found applications in surgical planning for jugular foramen tumors, treatment of cavernous dural arteriovenous fistulas, and evaluation of complex venous anatomy [11–13]. However, conventional RCV faces a fundamental technical limitation: contrast injection against the direction of physiological venous flow results in rapid washout and poor contrast retention, particularly in more proximal venous structures [5].

In the past, balloon catheters have been employed in cerebral venous interventions but focused primarily on navigation and device delivery rather than enhanced visualization [14–16]. We hypothesized that balloon occlusion during retrograde contrast injection would mechanically arrest downstream venous outflow, thereby increasing proximal pressure and improving retrograde contrast retention, thus enhancing visualization of cerebral venous structures upstream. This study aims to report a feasibility experience with balloon-assisted RCV (BARCV), comparing visualization quality of the cerebral venous architecture with and without balloon inflation to determine whether this technical modification addresses the fundamental limitations of conventional RCV.

## Materials and methods

### Study design and patient selection

We performed a retrospective study of consecutive patients who underwent endovascular procedures utilizing RCV between September 2023 and June 2025 at a single institution. Institutional review board approval was obtained prior to data collection, and all patients provided informed consent for their procedures. Inclusion criteria required availability of venographic images both before and after balloon inflation to enable paired comparison. Meanwhile, BARCV was not utilized in patients with no alternative non-dominant venous drainage channels.

### Procedural technique

**Fig. 1:**
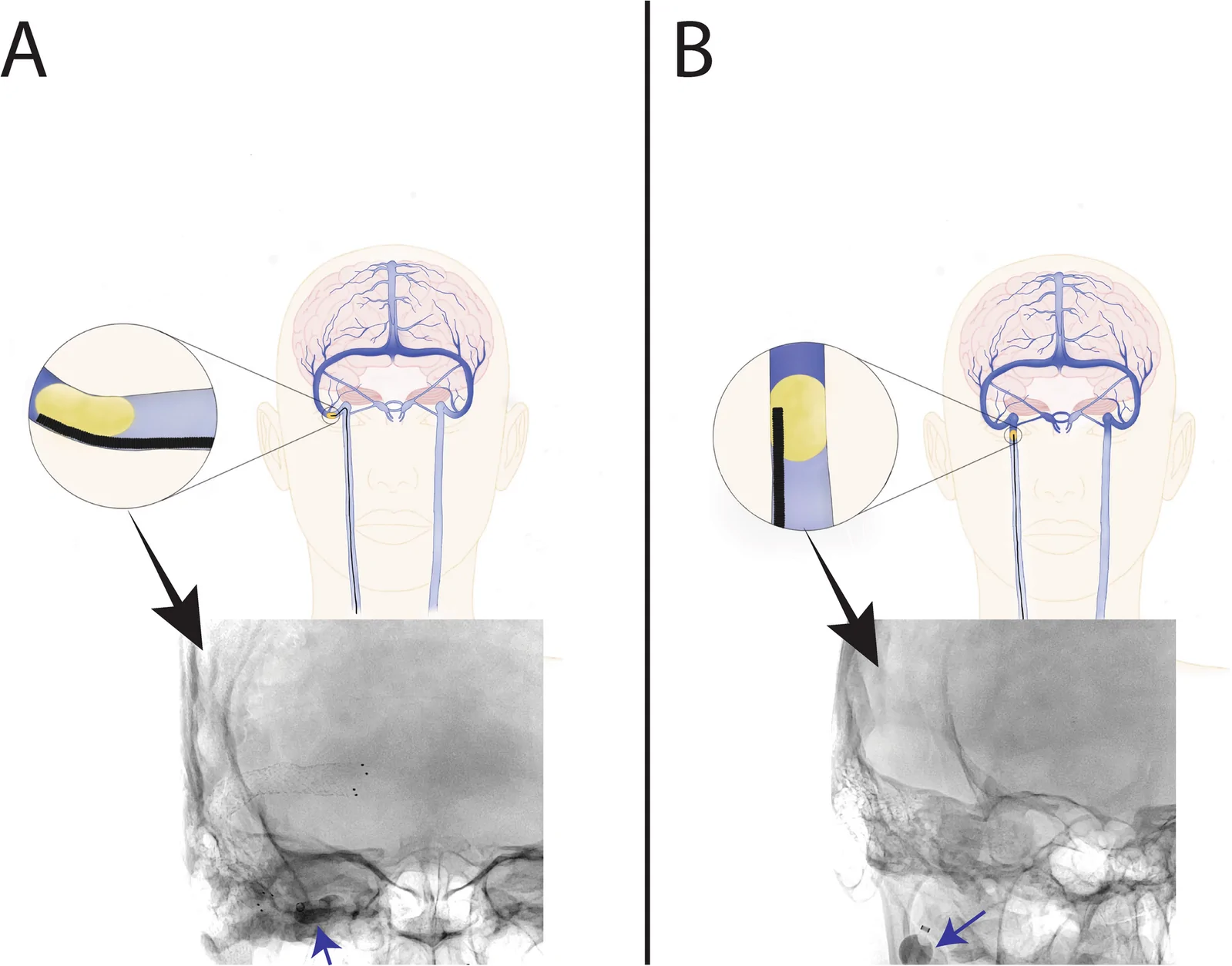
Illustration demonstrating the technique for retrograde balloon-assisted venography with inflation of a venous balloon guide catheter in the sigmoid sinus and jugular bulb, respectively. **A** Anteroposterior (AP) skull radiograph showing balloon inflation (blue arrow) in the right sigmoid sinus with retrograde opacification of dural venous sinuses demonstrated on the corresponding illustration. **B** AP skull radiograph showing balloon inflation (blue arrow) in the right internal jugular vein with retrograde opacification of dural venous sinuses demonstrated on the corresponding illustration

All procedures followed a standardized protocol and were performed with the patient in supine position under general anesthesia. Venous access was obtained via the cephalic vein, and a 9 French (9 F) short sheath was inserted. A 9 F Banner balloon guide catheter (EO Solutions Corp, Fort Lauderdale, Florida, USA) was advanced over a Glidewire Advantage (Terumo Corporation, Tokyo, Japan) and 5 F angled selection catheter (Penumbra, Alameda, California, USA) into the IJV or sigmoid sinus under fluoroscopic guidance (Fig. 1). A retrograde arterial 90% Isovue-300 (Bracco Diagnostics Inc., Princeton, NJ, USA) iodinated contrast injection was performed using an automated injector at standardized rates, namely 7 ml/sec for a total of 11 ml in case of common carotid artery injections, and 6 ml/sec for a total of 9 ml, in case of vertebral and internal carotid artery injections. Injections were performed at a rate of 15 pulses and 4 frames per second using the same angiography machine. Angiography was performed both before and after balloon inflation, not being performed in cases where adequate upstream opacification was attained without balloon inflation. A large field-of-view (FOV) was employed for all RCV acquisitions, with identical settings applied both before and after balloon inflation. Balloon inflation duration was maintained between 15 and 20 s in all cases and was followed by rapid deflation. Balloon inflation was only completed in cases with known bilateral dural venous sinus systems to ensure alternate venous drainage during the short duration of balloon inflation. Adequate collateral venous drainage was confirmed before inflation by restricting the technique to patients with a patent contralateral jugular vein and venous sinus system on non-invasive CTV/MRV; patients with an isolated or single dominant sinus were not selected for the technique. Where concomitant arterial access was available, robust venous clearance was confirmed on the venous phase of arteriography with the balloon inflated before retrograde injection. All procedures in this cohort were performed under general anesthesia, so neurological status could not be assessed clinically during the procedure. Vital signs were, however, monitored continuously and inflation was terminated rapidly at the end of the brief injection or sooner if any change in vital signs was observed. The short inflation time with rapid deflation was maintained in part to limit the risk associated with the absence of intraprocedural neurological assessment. Intracranial venous pressures were not recorded during inflation and therefore could not be evaluated in this retrospective cohort.

Concomitant cerebral arteriography was performed in all patients. Arterial access was obtained via the radial or ulnar artery under real-time ultrasound guidance using a 5 F sheath. Following arterial access, an intra-arterial cocktail consisting of verapamil (5 mg), nitroglycerin (200 µg), and heparin (2000 U) was administered. Selective angiography of the common and internal carotid arteries evaluated arterial inflow and venous outflow patterns during the venous phase.

## Data collection and image assessment

A standardized questionnaire was developed based on literature review and refined through input from five expert board certified neuroradiologists (Certificate of Added Qualification (CAQ) certified with 5–25 years of clinical experience) at our institution (Supplemental Data). Baseline parameters and procedural metrics included venous sinus dominance, mean IJV diameter measured at the C1 transverse process on DSA, balloon location, and maximum inflation diameter. Any cases of an incompletely occlusive balloon were identified by observing contrast filling downstream of the inflated balloon.

The primary outcome was visualization quality of the major venous sinuses (transverse sinus, sigmoid sinus, and transverse-sigmoid junction) and of the target lesion or pathology, compared with and without balloon inflation. Visualization of the remaining venous structures, together with contrast homogeneity, margin clarity, and the extent of opacification, were treated as a set of secondary and exploratory outcomes, reported in full in the supplementary tables. Visualization assessment encompassed multiple parameters. The most distant structure visible upstream of the IJV was recorded before and after balloon inflation. Specific evaluation of the lateral dural venous sinuses and ipsilateral condylar and emissary veins was performed. Ipsilateral cortical vein visualization was similarly documented. Pathology visualization quality was subjectively graded using a 3-point Likert scale (“Not very well,” “Somewhat well,” “Well”) before and after balloon inflation. Three board-certified neuroradiologists (CAQ certified with 5–25 years of clinical experience) independently evaluated anteroposterior and lateral view venograms involving a complete field of view (FOV) of the head, acquired at identical projections and imaging parameters, and viewed simultaneously on the Picture Archiving and Communication System (PACS). All reviewers remained blinded to the diagnosis, clinical outcomes, and other reviewers’ assessments during evaluation.

Additional clinical data were extracted from electronic medical records and procedural logs, including patient demographics (age, sex), procedural indication, radiation metrics (fluoroscopy time, radiation skin dose), procedure duration, hospital length of stay, and outcomes/complications.

### Statistical analysis

Continuous variables were reported as means with standard deviations. Categorical variables were reported as counts with percentages. McNemar’s test with continuity correction compared paired binary outcomes between conditions without and with balloon inflation. This non-parametric test was selected as appropriate for paired categorical data where each patient served as their own control. When insufficient discordant pairs precluded statistical testing, results were marked as not applicable. Two complementary effect size measures were used to assess the clinical meaningfulness of statistically significant findings. Odds ratios (OR) with 95% confidence intervals (CI) quantified the association between balloon inflation and each outcome. Cohen’s h provided a standardized effect size measure for differences between proportions, and was interpreted as small (0.2–0.5), medium (0.5–0.8) or large effect (> 0.8).

Inter-rater reliability among three independent reviewers was assessed using Cohen’s Kappa, calculated as the average of all pairwise Kappa values. Kappa values were interpreted according to Landis and Koch criteria: κ < 0.00 as poor agreement, κ = 0.00-0.20 as slight, κ = 0.21–0.40 as fair, κ = 0.41–0.60 as moderate, κ = 0.61–0.80 as substantial, and κ = 0.81-1.00 as almost perfect agreement. Cases of complete agreement indicated perfect reliability and precluded Kappa calculation due to zero variance.

All analyses were performed using Python 3.12 with SciPy (version 1.11.0), NumPy (version 1.24.0), pandas (version 2.0.0), and scikit-learn (version 1.3.0) libraries. A significance level of α = 0.05 was used for all hypothesis tests with two-tailed p-values.

## Results

### Baseline characteristics and procedural details

**Fig. 2:**
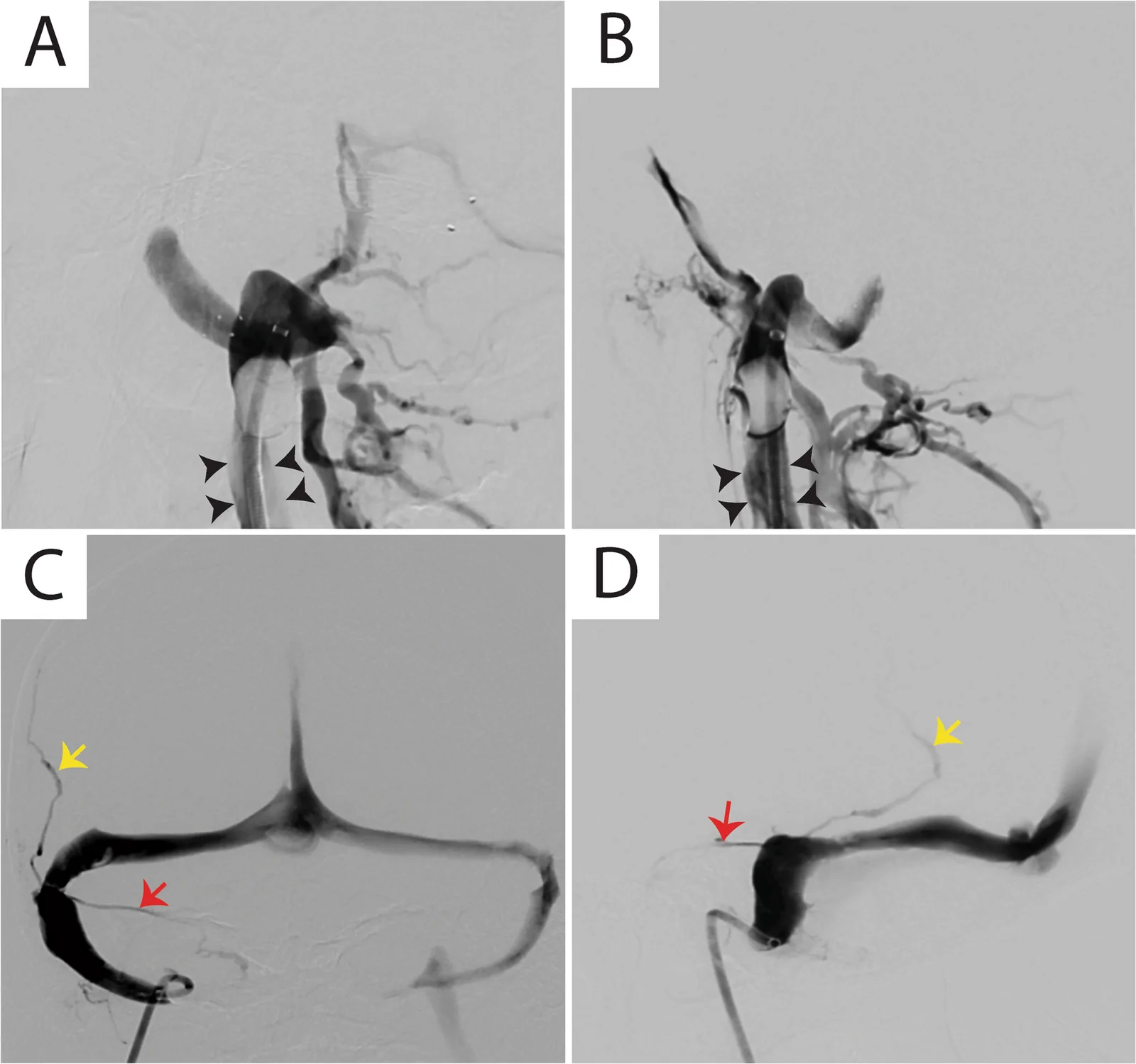
Retrograde Balloon Assisted Venography demonstrating two separate observed imaging features. **A&B** AP and lateral projection retrograde venogram showing balloon inflation with contrast filling downstream of the inflated balloon with slight filling of right internal jugular vein proximally (black arrowheads). This is seen when the inflated balloon guide is not completely occlusive secondary to large diameter and compliance of the jugular vein. **C&D** AP and lateral projection venogram with balloon occlusion demonstrating filling of bilateral transverse sigmoid sinuses and superior sagittal sinus with opacification of a right temporal cortical vein (yellow arrow) and right superior petrosal sinus (red arrow)

Seventeen patients underwent BARCV (Table 1), of whom 14 (82.4%) were female, with a mean age of 41.3 ± 15.2 years. Venous sinus dominance was right-sided in 10 patients (58.8%), left-sided in 3 (17.6%), and codominant in 4 (23.5%). Mean IJV diameter at the C1 transverse process was 10.23 ± 1.51 mm. The balloon was placed on the right in 11 patients (64.7%) and on the left in 6 (35.3%). Balloon location was the sigmoid sinus in 14 patients (82.4%) and the IJV in 3 (17.6%). Mean maximum inflated balloon diameter was 8.25 ± 2.33 mm with 4 cases (23.5%) noting an incompletely occlusive balloon due to small contrast filling downstream of it (Fig. 2). In each of these four cases, incomplete occlusion reflected a mismatch between the balloon and the venous diameter, as complete occlusion could not always be predicted before inflation. The mismatch was small, and only a minor amount of contrast passed proximal to the balloon, so upstream opacification was not meaningfully affected (Supplementary Fig. 1). Because upstream opacification was preserved in all four of these cases, they were not considered outliers, and their exclusion would not be expected to alter the direction of the primary findings. The primary indication was venous sinus stenting for stenosis in all 17 patients (100%). Nine patients (52.9%) underwent RCV for visualization during concomitant coil embolization of emissary veins or sinus diverticula.

**Table 1:** Demographics, baseline characteristics, and procedural details of balloon-occluded retrograde cerebral venography patients (*N* = 17)

Characteristic	Value
Demographics	
Age (years), mean ± SD	41.3 ± 15.2
Male, n (%)	3 (17.6%)
Female, n (%)	14 (82.4%)
Clinical Indication	
Pulsatile Tinnitus with venous stenosis, n (%)	15 (88.2%)
IIH with venous stenosis, n (%)	2 (11.8%)
Laterality of Stenosis	
Right-sided, n (%)	9 (52.9%)
Left-sided, n (%)	6 (35.3%)
Bilateral, n (%)	2 (11.8%)
Dominant venous system	
Left side, n (%)	3 (17.6%)
Right side, n (%)	10 (58.8%)
Codominant, n (%)	4 (23.5%)
Internal jugular vein diameter at C1 (mm), mean ± SD	10.23 ± 1.51
Balloon placement side	
Left, n (%)	6 (35.3%)
Right, n (%)	11 (64.7%)
Balloon location	
Internal jugular vein, n (%)	3 (17.6%)
Sigmoid sinus, n (%)	14 (82.4%)
Maximum inflated balloon diameter (mm), mean ± SD	8.25 ± 2.33
Incomplete balloon occlusion	
Yes, n (%)	4 (23.5%)
No, n (%)	13 (76.5%)
Primary Procedure	
Venous sinus stenting, n(%)	8 (47.1%)
Venous stenting + emissary vein/diverticulum coiling, n (%)	9 (52.9%)
Procedural Details	
Access via right cephalic vein, n (%)	17 (100%)
Fluoroscopy duration (minutes), mean ± SD (range)	39.7 ± 18.0 (15.0–81.5)
Skin radiation dose (mGy), mean ± SD (range)	1050.7 ± 908.5 (289–3559)
Outcomes	
Complications, n (%)	0 (0%)

### Impact of balloon inflation on pathology visualization

**Fig. 3:**
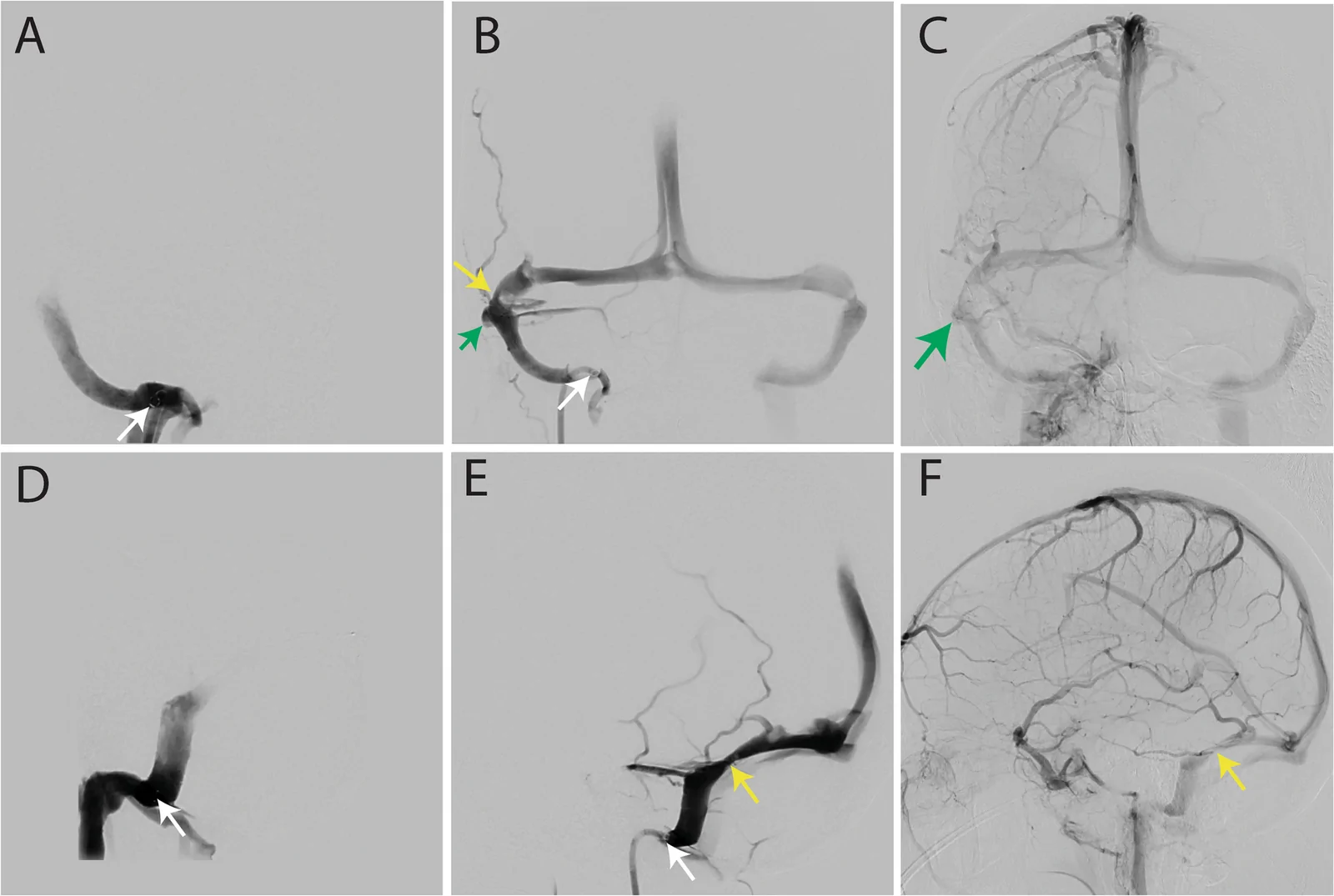
Venous Angiography in a 52-year-old male patient with right sided pulsatile tinnitus secondary to a right transverse-sigmoid junction stenosis and associated diverticulum. **A&D** Retrograde venography prior to balloon inflation (tip of balloon catheter demonstrated by white arrow) in right sigmoid sinus, shown on AP (**A**) & lateral (**D**) subtracted angiographic views with opacification of the right sigmoid sinus, jugular bulb, and associated condylar vein. **B&E** Corresponding AP and lateral subtracted retrograde venography views, respectively, after balloon inflation (white arrow) in the right sigmoid sinus showing significantly improved upstream opacification and visualization of venous stenosis (yellow arrow) and venous diverticulum (green arrow). **C**&**F** AP and lateral venous phase subtracted cerebral angiography views showing cortical venous anatomy and less conspicuous dural venous sinus anatomy compared to corresponding retrograde balloon assisted venography imaging (**B&E**). The right transverse sigmoid stenosis (yellow arrow) and right sinus venous diverticulum (green arrow) are identified

**Fig. 4:**
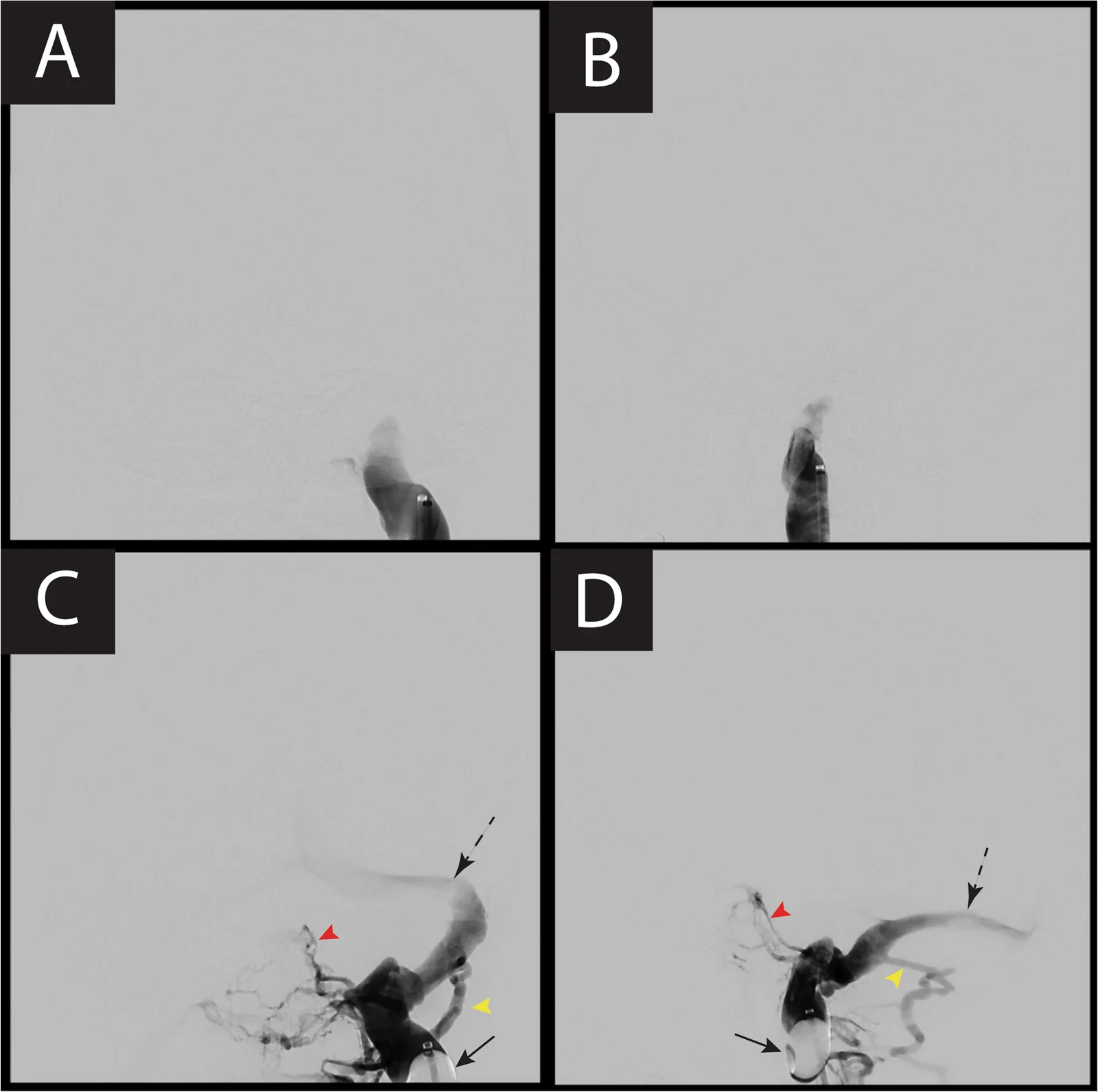
Retrograde venography in a 62-year-old female patient with left-sided pulsatile tinnitus and balloon placement in the left internal jugular vein (IJV). **A&B** AP and lateral subtracted angiographic views, respectively, showing retrograde venography from the left IJV with visualization of the left jugular vein and left jugular bulb. **C&D** Corresponding AP and lateral subtracted images, respectively, of retrograde left IJV venography with balloon inflation (black arrow) in the left IJV, demonstrating significantly improved upstream opacification of the left sigmoid sinus, left emissary vein (yellow arrowhead), and left inferior petrosal sinus (red arrowhead). A narrowing is noted at the left transverse-sigmoid junction secondary to stenosis (black dashed arrow)

Balloon inflation dramatically improved pathology visualization (Table 2, S1-S3; Figs. 3 and 4). Cases rated as “well visualized” increased from 15.7% to 88.2% (*p* < 0.001, OR = 29.04, 95% CI: 4.78-176.51, h = 1.627), while those rated “not well visualized” decreased significantly (*p* < 0.001, OR = 0.04, 95% CI: 0.00-0.44).

**Table 2:** Effect sizes and odds ratios comparing balloon inflation vs. no balloon (*N* = 17)

Outcome	Category	Without Balloon	With Balloon	Odds Ratio	95% CI	Cohen’s h	Effect Size
Ipsilateral/Midline Venous Structure Visibility							
Transverse sinus	Structure Visibility	35.3%	100.0%	61.92	3.17-1208.40	1.806	Large
Torcula	Structure Visibility	15.7%	86.3%	25.07	4.33-145.07	1.568	Large
Transverse-sigmoid junction	Structure Visibility	52.9%	100.0%	31.32	1.62-604.07	1.449	Large
Cortical veins	Structure Visibility	7.8%	58.8%	12.35	2.06–74.09	1.180	Large
Bridging veins	Structure Visibility	27.5%	43.1%	1.91	0.48–7.63	0.330	Small
Superior sagittal sinus	Structure Visibility	3.9%	47.1%	12.91	1.59-104.61	1.113	Large
Superior petrosal sinus	Structure Visibility	9.8%	33.3%	3.81	0.68–21.30	0.594	Medium
Sigmoid sinus	Structure Visibility	94.1%	100.0%	3.18	0.12–83.77	0.427	Small
Contralateral Venous Structure Visibility							
Transverse sinus	Structure Visibility	15.7%	90.2%	34.23	5.29-221.56	1.690	Large
Transverse-sigmoid junction	Structure Visibility	15.7%	78.4%	15.55	3.03–79.74	1.361	Large
Sigmoid sinus	Structure Visibility	15.7%	72.5%	11.63	2.38–56.82	1.224	Large
Bridging veins	Structure Visibility	0%	15.7%	7.47	0.35–159.65	0.751	Medium
Extent of Visualization							
Most distant landmark - contralateral structures	Extent of Visualization	15.7%	92.2%	41.31	5.87-290.53	1.759	Large
Dural Venous Sinus Quality							
Furthest visualization - torcula or beyond	Dural Sinus Quality	15.7%	86.3%	25.07	4.33-145.07	1.568	Large
Clear margins - both sinuses	Dural Sinus Quality	15.7%	80.4%	17.31	3.30-90.77	1.410	Large
Homogenous opacification - both sinuses	Dural Sinus Quality	13.7%	76.5%	16.06	3.06–84.25	1.370	Large
Condylar/Emissary veins quality							
Both veins - homogenous opacification	Condylar/Emissary Quality	19.6%	64.7%	6.54	1.48–28.80	0.952	Large
Both veins - clear margins	Condylar/Emissary Quality	21.6%	64.7%	5.87	1.37–25.23	0.903	Large
Both veins - furthest visualization	Condylar/Emissary Quality	25.5%	68.6%	5.68	1.35–23.88	0.894	Large
Pathology Assessment							
Pathology visualization rated “well”	Pathology Assessment	15.7%	88.2%	29.04	4.78-176.51	1.627	Large
Pathology visualization rated “not well” (decrease)	Pathology Assessment	54.9%	2.0%	0.04	0.00-0.44	1.388	Large

Odds ratios with Haldane correction. Effect size: Small (0.2–0.5), Medium (0.5–0.8), Large (> 0.8)

### Impact of balloon inflation on venous anatomical visualization

Without balloon inflation, the most common distal landmark was the ipsilateral sigmoid sinus (7.7 patients, 45.1%). With balloon inflation, this extended to the contralateral sigmoid sinus in 72.5% of cases versus 15.7% without (*p* < 0.001).

The detailed regional visualization of venous anatomy is stated below with patient counts representing averages across three independent reviewers. 

#### Major posterior and lateral dural venous sinuses 

**Fig. 5:**
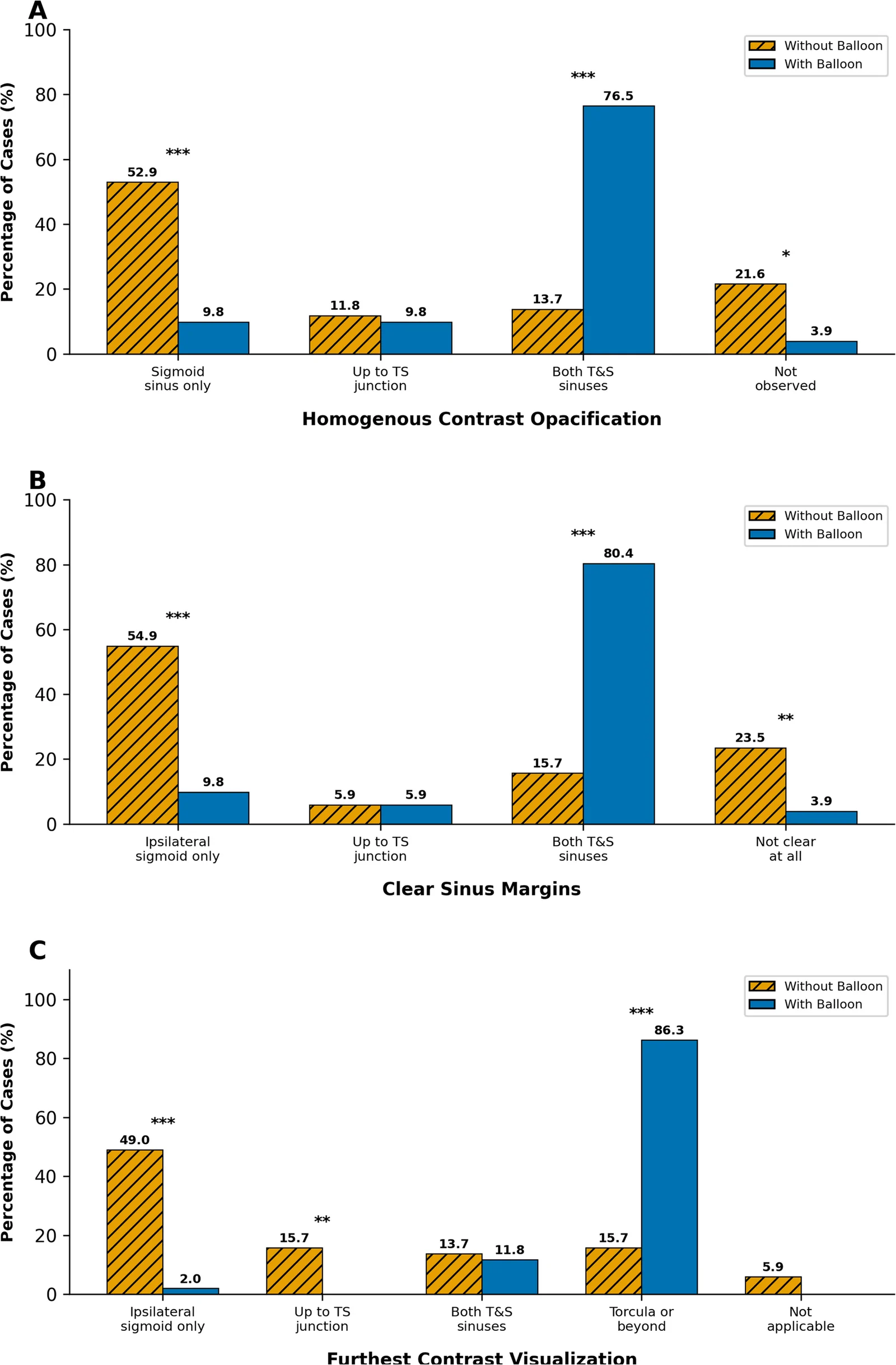
Comparison of Ipsilateral Lateral Dural Venous Sinus Visualization Quality Without vs. With Balloon Inflation. **A** Homogenous contrast opacification. With balloon: sigmoid sinus only 9.8%, up to transverse-sigmoid junction 9.8%, both transverse and sigmoid sinuses 76.5%, not observed 3.9%. Without balloon: sigmoid sinus only 52.9%, up to transverse-sigmoid junction 11.8%, both transverse and sigmoid sinuses 13.7%, not observed 21.6%. Significant differences: sigmoid sinus only (*p* < 0.001), both transverse and sigmoid sinuses (*p* < 0.001), not observed (*p* < 0.05). Non-significant: up to transverse-sigmoid junction (ns). *n* = 17 cases. **B** Clear sinus margins. With balloon: ipsilateral sigmoid only 9.8%, up to transverse-sigmoid junction 5.9%, both transverse and sigmoid sinuses 80.4%, not clear at all 3.9%. Without balloon: ipsilateral sigmoid only 54.9%, up to transverse-sigmoid junction 5.9%, both transverse and sigmoid sinuses 15.7%, not clear at all 23.5%. Significant differences: ipsilateral sigmoid only (*p* < 0.001), both transverse and sigmoid sinuses (*p* < 0.001), not clear at all (*p* < 0.01). Non-significant: up to transverse-sigmoid junction (ns). *n* = 17 cases. **C** Furthest contrast visualization. With balloon: ipsilateral sigmoid only 2.0%, up to transverse-sigmoid junction 0.0%, both transverse and sigmoid sinuses 11.8%, torcula or beyond 86.3%, not applicable 0.0%. Without balloon: ipsilateral sigmoid only 49.0%, up to transverse-sigmoid junction 15.7%, both transverse and sigmoid sinuses 13.7%, torcula or beyond 15.7%, not applicable 5.9%. Significant differences: ipsilateral sigmoid only (*p* < 0.001), up to transverse-sigmoid junction (*p* < 0.01), torcula or beyond (*p* < 0.001). Non-significant: both transverse and sigmoid sinuses (ns), not applicable (ns). *n* = 17 cases. Values represent the percentage of cases (*n* = 17). Statistical significance by McNemar’s test: *p < 0.05, **p < 0.01, ***p < 0.001, ns = not significant (*p* ≥ 0.05), N/A = not applicable (insufficient discordant pairs). Abbreviations: n, number of patients; ns, not significant; N/A, not applicable; T&S, transverse and sigmoid; TS, transverse-sigmoid

The ipsilateral transverse sinus, visible in only 35.3% of cases without balloon (κ = 0.829), achieved 100% visualization with balloon inflation (*p* < 0.001, OR = 61.92, Cohen’s h = 1.806). Likewise, visualization of the transverse-sigmoid junction improved from 52.9% to 100.0% (*p* < 0.001, OR = 31.32, h = 1.45). In contrast, the ipsilateral sigmoid sinus demonstrated excellent baseline visualization (94.1%) with minimal additional improvement (100.0%) with balloon inflation (*p* = 0.25). On the contralateral side, visualization likewise improved for the transverse sinus (*p* < 0.001, OR = 34.23, h = 1.69), transverse-sigmoid junction (*p* < 0.001, OR = 15.55, h = 1.36), and sigmoid sinus (*p* < 0.001, OR = 11.63, h = 1.22). Both homogenous opacification and clear margin delineation of the ipsilateral transverse and sigmoid sinuses improved markedly - from 13.7% to 76.5% and from 15.7% to 80.4%, respectively (both *p* < 0.001, OR = 16.06 and 17.31; Tables 2 and 3, S3-S4, S7, Fig. 5).

**Table 3:** Comparison of ipsilateral lateral dural venous sinus visualization quality without vs. with balloon inflation (*N* = 17)

Assessment Category	Without Balloon	With Balloon	Absolute Difference	*P*-value
Homogenous Contrast Opacification				
Both transverse and sigmoid sinuses	2.3 (13.7%)	13.0 (76.5%)	+ 62.7%	< 0.001***
Homogenous opacification not observed anywhere	3.7 (21.6%)	0.7 (3.9%)	-17.6%	0.012*
Sigmoid sinus only	9.0 (52.9%)	1.7 (9.8%)	-43.1%	< 0.001***
Up till the transverse-sigmoid junction	2.0 (11.8%)	1.7 (9.8%)	-2.0%	1.000
Clear Sinus Margins				
Both ipsilateral transverse and sigmoid sinuses	2.7 (15.7%)	13.7 (80.4%)	+ 64.7%	< 0.001***
Ipsilateral sigmoid sinus only	9.3 (54.9%)	1.7 (9.8%)	-45.1%	< 0.001***
Not clear at all	4.0 (23.5%)	0.7 (3.9%)	-19.6%	0.006**
Up till the ipsilateral transverse-sigmoid junction	1.0 (5.9%)	1.0 (5.9%)	+ 0.0%	1.000
Furthest Contrast Visualization				
Both ipsilateral transverse and sigmoid sinuses	2.3 (13.7%)	2.0 (11.8%)	-2.0%	1.000
Ipsilateral sigmoid sinus only	8.3 (49.0%)	0.3 (2.0%)	-47.1%	< 0.001***
Not applicable	1.0 (5.9%)	0.0 (0.0%)	-5.9%	0.250
Torcula or beyond	2.7 (15.7%)	14.7 (86.3%)	+ 70.6%	< 0.001***
Up till the ipsilateral transverse-sigmoid junction	2.7 (15.7%)	0.0 (0.0%)	-15.7%	0.008**

#### Other venous sinuses 

Visualization of the ipsilateral superior petrosal sinus (SPS) improved from 9.8% to 33.3% (*p* = 0.004, OR = 3.81, h = 0.59), while that of the inferior petrosal and cavernous sinuses remained unchanged. Visualization of the superior sagittal sinus improved significantly with balloon inflation (*p* < 0.001, OR = 12.91, h = 1.113), and contrast extending to the torcula or beyond increased from 15.7% to 86.3% (*p* < 0.001, OR = 25.07, h = 1.568). On the contralateral side, there was no improvement in visualization of the cavernous sinus or the superior and inferior petrosal sinuses (Tables S5, S6). 

#### Condylar and emissary veins 

Visualization extending to both condylar and emissary veins increased from 4.3 patients (25.5%) to 11.7 patients (68.6%) with balloon inflation (*p* < 0.001, OR = 5.68, h = 0.894). Homogenous opacification of both condylar and emissary veins increased from 19.6% to 64.7% (*p* < 0.001, OR = 6.54, h = 0.952). Clear margin delineation similarly improved from 21.6% to 64.7% (*p* < 0.001, OR = 5.87, h = 0.903; Tables S8, S9). 

#### Cortical and bridging veins

Ipsilateral cortical veins became visible in 10.0 patients (58.8%) compared to only 1.33 patients (7.8%) without balloon (*p* < 0.001, OR = 12.35, h = 1.180). With balloon occlusion, visualization of both ipsilateral and contralateral bridging veins also improved significantly - from 27.5% to 43.1%, and from 0% to 15.7%, respectively (*p* = 0.039 and 0.008, OR = 1.91 and 7.47, h = 0.330 and 0.751; Table 2, S4, Fig. 2).

### Safety outcomes

No procedure-related complications were observed in this limited cohort. All patients tolerated balloon inflation without adverse events during the brief inflation period. Given the small sample size, this absence of observed complications should not be taken as evidence that the technique is definitively safe.

### Inter-rater reliability

Agreement was substantial to almost perfect for key variables, including most distant landmark with balloon (κ = 0.728), ipsilateral transverse sinus without balloon (κ = 0.829), and ipsilateral sigmoid sinus with balloon (κ = 1.000). However, there was poor agreement for visualizing the sigmoid sinus without balloon inflation (κ= -0.028) and poor to fair agreement for visualizing bridging and cortical veins with balloon inflation (κ= -0.012-0.320). Several of these low values reflect the kappa paradox: when a structure is visualized in nearly all or nearly none of the cases, such as the sigmoid sinus without balloon (visualized in 94.1% of cases), near-uniform ratings constrain κ despite high observed agreement. Additionally, quality assessments pertaining to ‘homogenous opacification’ and ‘margins delineation’ of structures demonstrated only slight to fair agreement (κ = 0.156–0.344). The detailed inter-rater reliability values can be found in Table S3.

## Discussion

This study provides foundational evidence depicting the feasibility of BARCV in significantly improving visualization of the posterior and lateral dural venous sinuses and associated pathology compared with conventional RCV. With incomplete balloon occlusion occurring in only four cases, balloon inflation dramatically improved the quality and extent of venous visualization from the ipsilateral sigmoid sinus without balloon inflation to the contralateral sigmoid sinus with balloon inflation.

### Clinical applications and implications

Clear margin delineation improved from 15.7% to 80.4% in the transverse and sigmoid sinuses with balloon inflation. Improved delineation of venous sinus margins, together with more homogenous opacification, may in turn facilitate stent sizing, particularly at the commonly stented anatomically variable transverse-sigmoid junction, where undersizing or oversizing can cause complications. However, these findings are based on subjective visualization assessments made by experienced neuroradiologists without direct measurement of vessel diameters. Therefore, future studies are required to validate our findings by objectively correlating sinus diameter and stent size in the context of BARCV [6, 17].

Second, the technique’s ability to visualize bilateral venous anatomy from a unilateral access point may have important clinical implications. Considering 94% of patients with idiopathic intracranial hypertension have bilateral venous stenosis, this finding underscores BARCV’s diagnostic utility for visualizing contralateral pathology. This is particularly valuable when retrograde access via the contralateral side is precluded by a hypoplastic or thrombosed IJV [18–20]. In addition, the visualization of proximal venous anatomy through BARCV provides a safe roadmap to track the catheters and wires upstream, without necessarily requiring a venous map from a concomitant arterial access. This has the potential of simplifying & making venous navigation safer through superior visualization, thereby preventing cortical venous injury through blind wire navigation in the venous system [21].

Third, pathology was “well visualized” in 88.2% of cases with balloon as compared to 15.7% of cases without balloon; this demonstrates improved diagnostic confidence in not only visualizing venous sinus stenosis, but also concomitant sinus diverticula and prominent emissary veins. These early findings mandate confirmation in larger cohorts with a more diverse set of pathologies. However, this improved visualization may assist in selecting an appropriate stent size and in guiding stents or coils while avoiding potential venous complications [21].

Moreover, the better visualization of condylar and emissary veins with balloon inflation serves to suggest the role of BARCV in simulating transient sinus occlusion to reveal collateral venous pathways. This is in fact the deciding factor in assessing feasibility of stenting a sinus if we are to avoid high intracranial pressures or hemorrhage post-operatively [22, 23].

### Cortical vein visualization and safety considerations

There was a substantial increase in visualization of ipsilateral cortical veins (from 7.8% to 58.8%) with the balloon-assisted technique (Fig. 2). While this serves to reinforce better proximal visualization with balloon-inflation, it also cautions reflux leading to non-physiologic pressurization of cortical vein- an iatrogenic mimic of cortical venous reflux [24]. Prolonged balloon occlusion may cause an acute increase in intracranial venous pressure, potentially leading to cortical venous reflux, venous congestion, thrombosis, vessel injury, or hemorrhage [25, 26]. However, the reviewers demonstrated weak inter-rater reliability with respect to visualizing bridging and cortical veins after balloon inflation. This is an important point to remark upon given that despite the increase in cortical vein visualization, the potential associated complications were not noticed in any of our patients. Given this observation, a short duration of balloon inflation (15 to 20 s) must be emphasized, while ensuring an adequate total volume and controlled rate of contrast injection. We also ensured that this technique was employed in patients with intact bilateral venous systems based on non-invasive imaging.

Additionally, while proximal visualization of cortical veins necessitates caution, our study’s results resonate with the safe use of venous balloons in transjugular balloon thrombectomy procedures (OR: 273.94, 95% CI [63.77–1176.78]) [27]. Furthermore, considering the significant opacification of condylar veins with BARCV, transient balloon occlusion in the supine position essentially mimics normal physiological collapse of the IJV in the upright state whereby venous drainage is “siphoned” towards the vertebral venous plexus [28].

Regardless, this was only a feasibility study and does not eradicate safety concerns associated with potential over-pressurization of the intracranial venous system in patients with preexisting idiopathic intracranial hypertension (IIH). In fact, keeping patients’ safety into perspective, BARCV was not utilized in patients with no alternative non-dominant venous drainage channels. Future well-powered studies remain critical to further evaluating and establishing the safety of this technique.

### Integration with conventional angiography

Our initial experience with BARCV indicates that while the technique provides unparalleled anatomic detail of dural venous sinuses, skull base channels, and proximal bridging vein entry sites, it should not be used as a tool to visualize the deep venous system, cortical veins and bridging veins. DSA serves as the hemodynamic roadmap for parenchymal phase, cortical venous anatomy and deep cerebral venous system, while BARCV helps refine structural detail of the more distal dural venous sinus and jugular venous anatomy. Together, these techniques provide a comprehensive depiction of cerebral venous architecture: BARCV may enhance the precision of venous interventions, particularly in stent planning and sinus evaluation, while DSA preserves the physiological context necessary for safe and effective treatment. In these same patients, the venous phase obtained during carotid or vertebral arteriography provided substantially less detail of the dural venous sinuses than BARCV, because contrast is diluted during transit through the arterial circulation before reaching the venous phase (Fig. 3). BARCV is therefore best positioned as a focused adjunct to conventional angiography for detailed assessment of the dural venous sinuses, and not as a replacement for the physiological information that the venous phase of DSA provides.

### Comparison to existing techniques

Although DSA remains the gold standard for cerebrovascular imaging, delayed contrast washout in the venous phase limits its ability to adequately visualize cerebral venous anatomy. In a series of 89 patients, invasive IJV venography depicted superiority to static imaging in that it revealed dynamic stenotic changes not otherwise apparent on computed tomography (CT) or magnetic resonance imaging (MRI) [29]. The literature describes both antegrade dural sinus venography and conventional retrograde contrast injection in the IJV, though both techniques are limited by poor contrast retention [30, 31]. Our technique addresses these limitations through central balloon occlusion, which mechanically prevents rapid venous drainage and enables superior contrast accumulation in distal venous structures.

Past applications of balloon catheters in venous interventions have focused upon the use of Copernic balloons (BALT Extrusion, Montmorency, France) for transvenous embolization of cerebral arteriovenous malformations and to guide stents, including flow-diverting devices, via dual lumen balloon catheters [14, 15]. The Walrus balloon-guide catheter has similarly been used to navigate stents in venous sinus stenosis [16]. To our knowledge, however, balloon occlusion in the IJV or sigmoid sinus has not previously been evaluated specifically to enable proximal contrast entrapment and enhance venographic visualization of cerebral venous anatomy.

The hemodynamic principle underlying BARCV is not new. The original retrograde jugularography described by Gejrot and Laurén was performed under manual compression of the jugular vein distal to the catheter, and Quencer and colleagues emphasized that adequate neck compression, together with appropriate contrast volume and injection rate, is required to fill the intracranial dural sinuses and to avoid a falsely occlusive appearance [5, 10]. Manual compression arrests downstream jugular outflow and therefore reproduces, by external means, the same flow arrest that the balloon achieves intraluminally. BARCV may therefore be best understood as a catheter-based modernization of this established compression technique. However, several features distinguish the balloon approach; occlusion is controlled, complete, and reproducible, and does not depend on operator hand strength or on patient neck habitus, both of which constrain external compression. The balloon can also be positioned precisely within the sigmoid sinus, above the jugular bulb, a level that external neck compression cannot reach, which allows more targeted entrapment of contrast within the sinuses of interest. External compression is moreover applied to the internal jugular vein in the neck, whereas the balloon can be sited more distally within the sigmoid sinus, so the two approaches arrest venous outflow at different levels; in the small number of cases in which the balloon was instead inflated within the jugular vein, downstream contrast filling was more frequent, supporting the sigmoid sinus as the preferred balloon position (Fig. 2). Because the occlusion is maintained by the inflated balloon, the operator’s hands are free during injection and imaging, and the operator avoids the radiation exposure associated with holding manual compression in the field. Even heavy lead gloves attenuate but do not eliminate this dose, and they leave the hands within or adjacent to the primary beam. Manual compression, by contrast, is immediately available and carries no device cost. Our study did not compare BARCV directly against manual compression, so whether the additional visualization justifies an intraluminal device, and in which patients, remains to be established in a dedicated comparative study.

### Limitations & future directions

This study has some important limitations. While a feasibility study, the small sample size of 17 patients restricted us from performing subgroup analyses across different balloon inflation sites. The retrospective design and single-center experience may also introduce selection bias and limit applicability to other practice settings. In particular, when adequate upstream opacification was already achieved without balloon inflation, post-inflation acquisitions were not always obtained, so cases with favorable baseline visualization were under-represented in the paired comparison; this may exaggerate the apparent magnitude of the improvement attributed to balloon inflation. Additionally, all patients primarily underwent venous sinus stenting for stenosis, limiting generalizability to other pathologies. Furthermore, all patients in our study underwent general anesthesia; this may potentially limit the extension of our findings to cases performed with moderate or minimal sedation, although venous balloon occlusion testing for pulsatile tinnitus has reportedly remained well-tolerated in awake patients [32, 33]. Importantly, although the questionnaire was designed to be as objective as possible, reviewers’ image interpretations remained subjective. This is evident from the weaker inter-rater reliability values noted where “homogenous contrast opacification” and “clear margins delineation” needed to be commented upon to assess visualization quality. In addition, because the inflated balloon and the accompanying increase in opacification are visible on the venograms, reviewers could not be blinded to whether a given acquisition was obtained with or without balloon inflation, and this absence of condition blinding may bias the subjective visualization assessments in favor of the balloon-assisted images. We also performed a large number of statistical comparisons without formal correction for multiple testing, and the secondary and exploratory findings should therefore be regarded as hypothesis-generating. Relatedly, each patient was assessed by three reviewers, and the paired significance tests did not formally account for the resulting within-patient clustering of correlated reads; the reported p-values and confidence intervals should be regarded as optimistic.

Notably, minimal contrast filling was observed downstream of the inflated balloon in 2 of the 3 cases (67%) with IJV balloon placement, compared to only 14.3% of cases with balloon placement in the sigmoid sinus. However, given the small number of IJV cases, this percentage should be interpreted with caution. While the mean maximum inflation diameter of 11.4 mm in the IJV was nearly identical to the mean diameter of the IJV at the C1 transverse process, the higher rate of incomplete balloon occlusion with IJV balloon placement may be attributable to the greater compliance of the vein as compared to the rigid, dura-encased sigmoid sinus (Fig. 2). By the same reasoning, extrinsic manual compression would be expected to collapse the compliant jugular vein more completely than an intraluminal balloon, whose radial expansion may instead distend it, further supporting inflation within the rigid sigmoid sinus rather than the jugular vein for this technique. This observation warrants future evaluation of alternative balloon designs, sizing strategies, or positioning techniques to minimize downstream contrast filling while ensuring adequate visualization and prioritizing safety. The high anatomic variability of the transverse and sigmoid sinuses, reported in 79% of cases, may additionally influence technique effectiveness [6] Furthermore, future studies should systematically evaluate how septations, blind pouches, and other anatomical variants affect BARCV performance, as these variations can create localized flow stagnation zones that may mimic or exaggerate balloon-related contrast retention [6, 34].

Additionally, although balloon inflation improved delineation of the dural venous sinus margins, venous structures have higher capacitance and if pressurized with downstream balloon occlusion, may potentially cause normal veins to look bigger, leading to stent oversizing. While future research needs to qualify the accuracy of this argument, the present study primarily aimed to assess only the feasibility of visualization improvement with BARCV.

Prospective, multicenter studies with larger sample sizes and diverse pathologies are needed to validate these early findings and establish optimal balloon inflation and contrast injection parameters. Additionally, cost-effectiveness analysis comparing BARCV to alternative imaging strategies would inform clinical adoption decisions, while long-term follow-up studies assessing whether improved visualization translates to better procedural outcomes and reduced complications would establish clinical value.

Despite these limitations, BARCV consistently enabled better visualization across multiple anatomical regions and pathology types. The large effect sizes observed for key outcomes strongly indicate the technique’s clinical utility beyond statistical significance alone.

## Conclusion

BARCV is technically feasible and, in comparison to conventional RCV, offers improved visualization of the posterior and lateral dural venous sinuses and associated pathology. Additionally, no procedure-related complications were observed in this limited cohort. These findings support selective use of BARCV as an adjunct to conventional DSA for evaluating venous pathologies and for performing procedures like venous sinus stenting that necessitate accurate visualization of the commonly stented transverse and sigmoid sinuses. However, since this was a small, retrospective, single-center feasibility study, it only demonstrates improved visualization and not established clinical benefit, with larger studies needed to confirm these findings and establish the safety of this technique.

## Supplementary Information

Supplementary Material 1. Figure 1: Venous Phase Angiograms and Venograms from all 17 cases including the four cases (asterisk) in which balloon occlusion was incomplete. In each case, a small amount of contrast filled the vein (blue arrow) proximal to the inflated balloon because of a mismatch between the balloon and the venous diameter. Upstream opacification of the dural venous sinuses was preserved in all four cases. Table S1: Comparison of Pathology Visualization Quality Without vs. With Balloon Inflation. Table S2: Pathology Visualization Quality Without and With Balloon Inflation by Individual Reviewer. Table S3: Inter-rater Reliability Analysis for All Major Study Variables. Table S4: Comparison of Venous Structure Visibility Upstream of the Internal Jugular Vein (IJV) Without vs. With Balloon Inflation. Table S5: Visibility of Venous Structures Upstream of the Internal Jugular Vein (IJV) Without and With Balloon Inflation. Table S6: Most Distant Landmark Visualized Upstream of the Internal Jugular Vein (IJV) Without and With Balloon Inflation. Table S7: Ipsilateral Lateral Dural Venous Sinus Visualization Quality Without and With Balloon Inflation. Table S8: Comparison of Ipsilateral Condylar and Emissary Veins Visualization Quality Without vs. With Balloon Inflation. Table S9: Ipsilateral Condylar and Emissary Veins Visualization Quality Without and With Balloon Inflation.

## Data Availability

No datasets were generated or analysed during the current study.

## Abbreviations

BARCV: Balloon-assisted retrograde cerebral venography
CAQ: Certificate of Added Qualification
CI: Confidence interval
CT: Computed tomography
CTV: Computed tomography venography
DSA: Digital subtraction angiography
FOV: Field of view
IIH: Idiopathic intracranial hypertension
IJV: Internal jugular vein
MRI: Magnetic resonance imaging
MRV: Magnetic resonance venography
OR: Odds ratio
PACS: Picture Archiving and Communication System
RCV: Retrograde cerebral venography
SPS: Superior petrosal sinus
