# Supplementary material for: Balloon-assisted retrograde cerebral venography for enhanced visualization of the posterior and lateral dural venous sinuses: a feasibility study
Source: Neuroradiology. 2026 Jul 10;68(8):2353–68. doi: 10.1007/s00234-026-04110-9 (PMC13577980; doi:10.1007/s00234-026-04110-9)
Supplement: Supplementary file 1 [file 234_2026_4110_MOESM1_ESM.docx]

**Supplementary Material**

*Supplementary Figure 1:* Venous Phase Angiograms and Venograms from all 17 cases including the four cases (asterisk) in which balloon occlusion was incomplete. In each case, a small amount of contrast filled the vein (blue arrow) proximal to the inflated balloon because of a mismatch between the balloon and the venous diameter. Upstream opacification of the dural venous sinuses was preserved in all four cases. *Table S1:* Comparison of Pathology Visualization Quality Without vs. With Balloon Inflation

*Table S2:* Pathology Visualization Quality Without and With Balloon Inflation by Individual Reviewer

*Table S3:* Inter-rater Reliability Analysis for All Major Study Variables

*Table S4:* Comparison of Venous Structure Visibility Upstream of the Internal Jugular Vein (IJV) Without vs. With Balloon Inflation

*Table S5:* Visibility of Venous Structures Upstream of the Internal Jugular Vein (IJV) Without and With Balloon Inflation

*Table S6:* Most Distant Landmark Visualized Upstream of the Internal Jugular Vein (IJV) Without and With Balloon Inflation

*Table S7:* Ipsilateral Lateral Dural Venous Sinus Visualization Quality Without and With Balloon Inflation

*Table S8:* Comparison of Ipsilateral Condylar and Emissary Veins Visualization Quality Without vs. With Balloon Inflation

*Table S9:* Ipsilateral Condylar and Emissary Veins Visualization Quality Without and With Balloon Inflation

*REDCap Survey*

**Supplementary Material**

*Supplementary Figure 1:* Venous Phase Angiograms and Venograms from all 17 cases including the four cases (asterisk) in which balloon occlusion was incomplete. In each case, a small amount of contrast filled the vein (blue arrow) proximal to the inflated balloon because of a mismatch between the balloon and the venous diameter. Upstream opacification of the dural venous sinuses was preserved in all four cases.

**Table S1.** Comparison of Pathology Visualization Quality Without vs. With Balloon Inflation (N=17 patients)

| **Quality Rating** | **Without Balloon** | **With Balloon** | **Absolute Difference** | **P-value** |
| --- | --- | --- | --- | --- |
| Well | 2.67 (15.7%) | 15.00 (88.2%) | +72.5% | <0.001*** |
| Somewhat well | 5.00 (29.4%) | 1.67 (9.8%) | -19.6% | 0.021* |
| Not well | 9.33 (54.9%) | 0.33 (2.0%) | -52.9% | <0.001*** |

**Note:** Patient counts represent averages across three independent reviewers. P-values from McNemar’s test. *p<0.05, **p<0.01, ***p<0.001.

**Table S2.** Pathology Visualization Quality Without and With Balloon Inflation by Individual Reviewer (N=17)

| **Quality Rating** | **Reviewer #1** | **Reviewer #2** | **Reviewer #3** | **Average** |
| --- | --- | --- | --- | --- |
| **WITHOUT BALLOON INFLATION, n (%)** |  |  |  |  |
| Well | 6 (35.3%) | 1 (5.9%) | 1 (5.9%) | 2.67 (15.7%) |
| Somewhat well | 5 (29.4%) | 5 (29.4%) | 5 (29.4%) | 5.00 (29.4%) |
| Not well | 6 (35.3%) | 11 (64.7%) | 11 (64.7%) | 9.33 (54.9%) |
| **WITH BALLOON INFLATION, n (%)** |  |  |  |  |
| Well | 16 (94.1%) | 16 (94.1%) | 13 (76.5%) | 15.00 (88.2%) |
| Somewhat well | 1 (5.9%) | 0 (0.0%) | 4 (23.5%) | 1.67 (9.8%) |
| Not well | 0 (0.0%) | 1 (5.9%) | 0 (0.0%) | 0.33 (2.0%) |

**Note:** Three independent reviewers assessed all cases.

**Table S3.** Inter-rater Reliability Analysis for All Major Study Variables (N=17)

| **Variable** | **Category** | **Cohen’s Kappa** | **Interpretation** |
| --- | --- | --- | --- |
| **STRUCTURE VISIBILITY - WITHOUT BALLOON** |  |  |  |
| Transverse sinus (ipsilateral) | Visibility | 0.829 | Almost Perfect |
| Transverse sinus (contralateral) | Visibility | 0.736 | Substantial |
| Transverse-sigmoid junction (ipsilateral) | Visibility | 0.372 | Fair |
| Transverse-sigmoid junction (contralateral) | Visibility | 0.736 | Substantial |
| Sigmoid sinus (ipsilateral) | Visibility | -0.028 | Poor |
| Sigmoid sinus (contralateral) | Visibility | 0.736 | Substantial |
| Torcula | Visibility | 0.736 | Substantial |
| Bridging veins (ipsilateral) | Visibility | 0.333 | Fair |
| Bridging veins (contralateral) | Visibility | 1.000 | Almost Perfect |
| Cortical veins | Visibility | 0.759 | Substantial |
| **STRUCTURE VISIBILITY - WITH BALLOON** |  |  |  |
| Transverse sinus (ipsilateral) | Visibility | 1.000 | Almost Perfect |
| Transverse sinus (contralateral) | Visibility | 0.634 | Substantial |
| Transverse-sigmoid junction (ipsilateral) | Visibility | 1.000 | Almost Perfect |
| Transverse-sigmoid junction (contralateral) | Visibility | 0.652 | Substantial |
| Sigmoid sinus (ipsilateral) | Visibility | 1.000 | Almost Perfect |
| Sigmoid sinus (contralateral) | Visibility | 0.703 | Substantial |
| Torcula | Visibility | 0.345 | Fair |
| Bridging veins (ipsilateral) | Visibility | -0.012 | Poor |
| Bridging veins (contralateral) | Visibility | 0.006 | Slight |
| Cortical veins | Visibility | 0.320 | Fair |
| **MOST DISTANT LANDMARK** |  |  |  |
| Most distant landmark (with balloon) | Assessment | 0.728 | Substantial |
| Most distant landmark (without balloon) | Assessment | 0.482 | Moderate |
| **DURAL VENOUS SINUS QUALITY** |  |  |  |
| Dural venous sinus - homogenous opacification (without balloon) | Quality | 0.200 | Slight |
| Clear margins (without balloon) | Quality | 0.344 | Fair |
| Dural venous sinus - homogenous opacification (with balloon) | Quality | 0.073 | Slight |
| Clear margins (with balloon) | Quality | 0.103 | Slight |
| **CONDYLAR/EMISSARY VEINS** |  |  |  |
| Condylar/emissary - homogenous opacification (without balloon) | Quality | 0.050 | Slight |
| Condylar/emissary - homogenous opacification (with balloon) | Quality | 0.110 | Slight |
| **PATHOLOGY VISUALIZATION** |  |  |  |
| Pathology visualization quality (without balloon) | Assessment | 0.309 | Fair |
| Pathology visualization quality (with balloon) | Assessment | 0.156 | Slight |

**Note:** Cohen’s Kappa calculated as average of all pairwise values. Interpretation per Landis and Koch.

**Table S4.** Comparison of Venous Structure Visibility Upstream of the IJV Without vs. With Balloon Inflation (N=17)

| **Anatomical Structure** | **Without Balloon** | **With Balloon** | **Absolute Difference** | **P-value** |
| --- | --- | --- | --- | --- |
| **Ipsilateral Structures** |  |  |  |  |
| Sigmoid sinus | 16.0 (94.1%) | 17.0 (100.0%) | +5.9% | 0.250 |
| Transverse-sigmoid junction | 9.0 (52.9%) | 17.0 (100.0%) | +47.1% | <0.001*** |
| Transverse sinus | 6.0 (35.3%) | 17.0 (100.0%) | +64.7% | <0.001*** |
| Superior petrosal sinus | 1.7 (9.8%) | 5.7 (33.3%) | +23.5% | 0.004** |
| Inferior petrosal sinus | 1.7 (9.8%) | 2.3 (13.7%) | +3.9% | 0.688 |
| Cavernous sinus | 1.3 (7.8%) | 2.3 (13.7%) | +5.9% | 0.375 |
| Bridging veins | 4.7 (27.5%) | 7.3 (43.1%) | +15.7% | 0.039* |
| Cortical veins | 1.3 (7.8%) | 10.0 (58.8%) | +51.0% | <0.001*** |
| Deep venous structures | 2.0 (11.8%) | 3.3 (19.6%) | +7.8% | 0.219 |
| **Midline Structures** |  |  |  |  |
| Torcula | 2.7 (15.7%) | 14.7 (86.3%) | +70.6% | <0.001*** |
| Superior sagittal sinus | 0.7 (3.9%) | 8.0 (47.1%) | +43.1% | <0.001*** |
| Straight sinus | 0.7 (3.9%) | 1.3 (7.8%) | +3.9% | 0.688 |
| Inferior sagittal sinus | 0.0 (0.0%) | 0.3 (2.0%) | +2.0% | 1.000 |
| **Contralateral Structures** |  |  |  |  |
| Sigmoid sinus | 2.7 (15.7%) | 12.3 (72.5%) | +56.9% | <0.001*** |
| Transverse-sigmoid junction | 2.7 (15.7%) | 13.3 (78.4%) | +62.7% | <0.001*** |
| Transverse sinus | 2.7 (15.7%) | 15.3 (90.2%) | +74.5% | <0.001*** |
| Superior petrosal sinus | 0.3 (2.0%) | 0.3 (2.0%) | +0.0% | 1.000 |
| Inferior petrosal sinus | 0.7 (3.9%) | 1.7 (9.8%) | +5.9% | 0.375 |
| Cavernous sinus | 0.7 (3.9%) | 1.0 (5.9%) | +2.0% | 1.000 |
| Bridging veins | 0.0 (0.0%) | 2.7 (15.7%) | +15.7% | 0.008** |
| Cortical veins | 0.0 (0.0%) | 0.0 (0.0%) | +0.0% | N/A |
| Deep venous structures | 0.3 (2.0%) | 1.0 (5.9%) | +3.9% | 0.625 |
| **Most Distant Landmark Visualized Upstream of IJV** |  |  |  |  |
| Contralateral transverse-sigmoid junction | 0.0 (0.0%) | 0.7 (3.9%) | +3.9% | 0.500 |
| Contralateral sigmoid sinus | 2.7 (15.7%) | 12.3 (72.5%) | +56.9% | <0.001*** |
| Contralateral transverse sinus | 0.0 (0.0%) | 2.7 (15.7%) | +15.7% | 0.008** |
| Ipsilateral sigmoid sinus | 7.7 (45.1%) | 0.0 (0.0%) | -45.1% | <0.001*** |
| Ipsilateral transverse sinus | 3.3 (19.6%) | 1.3 (7.8%) | -11.8% | 0.180 |
| Ipsilateral transverse-sigmoid junction | 2.3 (13.7%) | 0.0 (0.0%) | -13.7% | 0.016* |
| Not applicable | 1.0 (5.9%) | 0.0 (0.0%) | -5.9% | 0.250 |

**Note:** Patient counts represent averages across three reviewers. P-values from McNemar’s test. *p<0.05, **p<0.01, ***p<0.001.

**Table S5.** Visibility of Venous Structures by Individual Reviewer (N=17)

| **Anatomical Structure** | **Reviewer #1** | **Reviewer #2** | **Reviewer #3** | **Average** |
| --- | --- | --- | --- | --- |
| **WITHOUT BALLOON INFLATION** |  |  |  |  |
| **Ipsilateral Structures** |  |  |  |  |
| Sigmoid sinus | 16 (94.1%) | 15 (88.2%) | 17 (100.0%) | 16.00 (94.1%) |
| Transverse-sigmoid junction | 10 (58.8%) | 9 (52.9%) | 8 (47.1%) | 9.00 (52.9%) |
| Transverse sinus | 7 (41.2%) | 5 (29.4%) | 6 (35.3%) | 6.00 (35.3%) |
| Superior petrosal sinus | 2 (11.8%) | 2 (11.8%) | 1 (5.9%) | 1.67 (9.8%) |
| Inferior petrosal sinus | 2 (11.8%) | 1 (5.9%) | 2 (11.8%) | 1.67 (9.8%) |
| Cavernous sinus | 2 (11.8%) | 1 (5.9%) | 1 (5.9%) | 1.33 (7.8%) |
| Bridging veins | 0 (0.0%) | 14 (82.4%) | 0 (0.0%) | 4.67 (27.5%) |
| Cortical veins | 2 (11.8%) | 1 (5.9%) | 1 (5.9%) | 1.33 (7.8%) |
| Deep venous structures | 2 (11.8%) | 4 (23.5%) | 0 (0.0%) | 2.00 (11.8%) |
| **Midline Structures** |  |  |  |  |
| Torcula | 4 (23.5%) | 2 (11.8%) | 2 (11.8%) | 2.67 (15.7%) |
| Superior sagittal sinus | 1 (5.9%) | 1 (5.9%) | 0 (0.0%) | 0.67 (3.9%) |
| Straight sinus | 2 (11.8%) | 0 (0.0%) | 0 (0.0%) | 0.67 (3.9%) |
| Inferior sagittal sinus | 0 (0.0%) | 0 (0.0%) | 0 (0.0%) | 0.00 (0.0%) |
| **Contralateral Structures** |  |  |  |  |
| Sigmoid sinus | 4 (23.5%) | 2 (11.8%) | 2 (11.8%) | 2.67 (15.7%) |
| Transverse-sigmoid junction | 4 (23.5%) | 2 (11.8%) | 2 (11.8%) | 2.67 (15.7%) |
| Transverse sinus | 4 (23.5%) | 2 (11.8%) | 2 (11.8%) | 2.67 (15.7%) |
| Superior petrosal sinus | 1 (5.9%) | 0 (0.0%) | 0 (0.0%) | 0.33 (2.0%) |
| Inferior petrosal sinus | 2 (11.8%) | 0 (0.0%) | 0 (0.0%) | 0.67 (3.9%) |
| Cavernous sinus | 2 (11.8%) | 0 (0.0%) | 0 (0.0%) | 0.67 (3.9%) |
| Bridging veins | 0 (0.0%) | 0 (0.0%) | 0 (0.0%) | 0.00 (0.0%) |
| Cortical veins | 0 (0.0%) | 0 (0.0%) | 0 (0.0%) | 0.00 (0.0%) |
| Deep venous structures | 1 (5.9%) | 0 (0.0%) | 0 (0.0%) | 0.33 (2.0%) |
|  |  |  |  |  |
| **WITH BALLOON INFLATION** |  |  |  |  |
| **Ipsilateral Structures** |  |  |  |  |
| Sigmoid sinus | 17 (100.0%) | 17 (100.0%) | 17 (100.0%) | 17.00 (100.0%) |
| Transverse-sigmoid junction | 17 (100.0%) | 17 (100.0%) | 17 (100.0%) | 17.00 (100.0%) |
| Transverse sinus | 17 (100.0%) | 17 (100.0%) | 17 (100.0%) | 17.00 (100.0%) |
| Superior petrosal sinus | 2 (11.8%) | 9 (52.9%) | 6 (35.3%) | 5.67 (33.3%) |
| Inferior petrosal sinus | 2 (11.8%) | 2 (11.8%) | 3 (17.6%) | 2.33 (13.7%) |
| Cavernous sinus | 1 (5.9%) | 4 (23.5%) | 2 (11.8%) | 2.33 (13.7%) |
| Bridging veins | 0 (0.0%) | 15 (88.2%) | 7 (41.2%) | 7.33 (43.1%) |
| Cortical veins | 10 (58.8%) | 13 (76.5%) | 7 (41.2%) | 10.00 (58.8%) |
| Deep venous structures | 1 (5.9%) | 8 (47.1%) | 1 (5.9%) | 3.33 (19.6%) |
| **Midline Structures** |  |  |  |  |
| Torcula | 15 (88.2%) | 15 (88.2%) | 14 (82.4%) | 14.67 (86.3%) |
| Superior sagittal sinus | 8 (47.1%) | 5 (29.4%) | 11 (64.7%) | 8.00 (47.1%) |
| Straight sinus | 1 (5.9%) | 1 (5.9%) | 2 (11.8%) | 1.33 (7.8%) |
| Inferior sagittal sinus | 0 (0.0%) | 1 (5.9%) | 0 (0.0%) | 0.33 (2.0%) |
| **Contralateral Structures** |  |  |  |  |
| Sigmoid sinus | 11 (64.7%) | 14 (82.4%) | 12 (70.6%) | 12.33 (72.5%) |
| Transverse-sigmoid junction | 13 (76.5%) | 14 (82.4%) | 13 (76.5%) | 13.33 (78.4%) |
| Transverse sinus | 14 (82.4%) | 16 (94.1%) | 16 (94.1%) | 15.33 (90.2%) |
| Superior petrosal sinus | 0 (0.0%) | 0 (0.0%) | 1 (5.9%) | 0.33 (2.0%) |
| Inferior petrosal sinus | 1 (5.9%) | 1 (5.9%) | 3 (17.6%) | 1.67 (9.8%) |
| Cavernous sinus | 1 (5.9%) | 1 (5.9%) | 1 (5.9%) | 1.00 (5.9%) |
| Bridging veins | 0 (0.0%) | 4 (23.5%) | 4 (23.5%) | 2.67 (15.7%) |
| Cortical veins | 0 (0.0%) | 0 (0.0%) | 0 (0.0%) | 0.00 (0.0%) |
| Deep venous structures | 1 (5.9%) | 2 (11.8%) | 0 (0.0%) | 1.00 (5.9%) |

**Table S6.** Most Distant Landmark by Individual Reviewer (N=17)

| **Most Distant Landmark** | **Reviewer #1** | **Reviewer #2** | **Reviewer #3** | **Average** |
| --- | --- | --- | --- | --- |
| **WITHOUT BALLOON INFLATION, n (%)** |  |  |  |  |
| Contralateral sigmoid sinus | 4 (23.5%) | 2 (11.8%) | 2 (11.8%) | 2.67 (15.7%) |
| Ipsilateral sigmoid sinus | 7 (41.2%) | 7 (41.2%) | 9 (52.9%) | 7.67 (45.1%) |
| Ipsilateral transverse sinus | 3 (17.6%) | 3 (17.6%) | 4 (23.5%) | 3.33 (19.6%) |
| Ipsilateral transverse-sigmoid junction | 2 (11.8%) | 3 (17.6%) | 2 (11.8%) | 2.33 (13.7%) |
| Not applicable | 1 (5.9%) | 2 (11.8%) | 0 (0.0%) | 1.00 (5.9%) |
| **WITH BALLOON INFLATION, n (%)** |  |  |  |  |
| Contralateral transverse-sigmoid junction | 1 (5.9%) | 0 (0.0%) | 1 (5.9%) | 0.67 (3.9%) |
| Contralateral sigmoid sinus | 11 (64.7%) | 14 (82.4%) | 12 (70.6%) | 12.33 (72.5%) |
| Contralateral transverse sinus | 3 (17.6%) | 2 (11.8%) | 3 (17.6%) | 2.67 (15.7%) |
| Ipsilateral transverse sinus | 2 (11.8%) | 1 (5.9%) | 1 (5.9%) | 1.33 (7.8%) |

**Table S7.** Ipsilateral Lateral Dural Venous Sinus Visualization Quality by Individual Reviewer (N=17)

| **Assessment Category** | **Reviewer #1** | **Reviewer #2** | **Reviewer #3** | **Average** |
| --- | --- | --- | --- | --- |
| **WITHOUT BALLOON INFLATION** |  |  |  |  |
| **Homogenous Contrast Opacification, n (%)** |  |  |  |  |
| Both transverse and sigmoid sinuses | 4 (23.5%) | 0 (0.0%) | 3 (17.6%) | 2.33 (13.7%) |
| Homogenous opacification not observed anywhere | 2 (11.8%) | 3 (17.6%) | 6 (35.3%) | 3.67 (21.6%) |
| Sigmoid sinus only | 11 (64.7%) | 9 (52.9%) | 7 (41.2%) | 9.00 (52.9%) |
| Up till the transverse-sigmoid junction | 0 (0.0%) | 5 (29.4%) | 1 (5.9%) | 2.00 (11.8%) |
| **Clear Sinus Margins, n (%)** |  |  |  |  |
| Both ipsilateral transverse and sigmoid sinuses | 4 (23.5%) | 1 (5.9%) | 3 (17.6%) | 2.67 (15.7%) |
| Ipsilateral sigmoid sinus only | 10 (58.8%) | 10 (58.8%) | 8 (47.1%) | 9.33 (54.9%) |
| Not clear at all | 3 (17.6%) | 3 (17.6%) | 6 (35.3%) | 4.00 (23.5%) |
| Up till the ipsilateral transverse-sigmoid junction | 0 (0.0%) | 3 (17.6%) | 0 (0.0%) | 1.00 (5.9%) |
| **Furthest Contrast Visualization, n (%)** |  |  |  |  |
| Both ipsilateral transverse and sigmoid sinuses | 3 (17.6%) | 2 (11.8%) | 2 (11.8%) | 2.33 (13.7%) |
| Ipsilateral sigmoid sinus only | 8 (47.1%) | 8 (47.1%) | 9 (52.9%) | 8.33 (49.0%) |
| Not applicable | 1 (5.9%) | 2 (11.8%) | 0 (0.0%) | 1.00 (5.9%) |
| Torcula or beyond | 4 (23.5%) | 2 (11.8%) | 2 (11.8%) | 2.67 (15.7%) |
| Up till the ipsilateral transverse-sigmoid junction | 1 (5.9%) | 3 (17.6%) | 4 (23.5%) | 2.67 (15.7%) |
|  |  |  |  |  |
| **WITH BALLOON INFLATION** |  |  |  |  |
| **Homogenous Contrast Opacification, n (%)** |  |  |  |  |
| Both transverse and sigmoid sinuses | 13 (76.5%) | 14 (82.4%) | 12 (70.6%) | 13.00 (76.5%) |
| Homogenous opacification not observed anywhere | 0 (0.0%) | 0 (0.0%) | 2 (11.8%) | 0.67 (3.9%) |
| Sigmoid sinus only | 3 (17.6%) | 0 (0.0%) | 2 (11.8%) | 1.67 (9.8%) |
| Up till the transverse-sigmoid junction | 1 (5.9%) | 3 (17.6%) | 1 (5.9%) | 1.67 (9.8%) |
| **Clear Sinus Margins, n (%)** |  |  |  |  |
| Both ipsilateral transverse and sigmoid sinuses | 14 (82.4%) | 15 (88.2%) | 12 (70.6%) | 13.67 (80.4%) |
| Ipsilateral sigmoid sinus only | 3 (17.6%) | 0 (0.0%) | 2 (11.8%) | 1.67 (9.8%) |
| Not clear at all | 0 (0.0%) | 0 (0.0%) | 2 (11.8%) | 0.67 (3.9%) |
| Up till the ipsilateral transverse-sigmoid junction | 0 (0.0%) | 2 (11.8%) | 1 (5.9%) | 1.00 (5.9%) |
| **Furthest Contrast Visualization, n (%)** |  |  |  |  |
| Both ipsilateral transverse and sigmoid sinuses | 1 (5.9%) | 1 (5.9%) | 4 (23.5%) | 2.00 (11.8%) |
| Ipsilateral sigmoid sinus only | 0 (0.0%) | 0 (0.0%) | 1 (5.9%) | 0.33 (2.0%) |
| Torcula or beyond | 16 (94.1%) | 16 (94.1%) | 12 (70.6%) | 14.67 (86.3%) |

**Table S8.** Comparison of Ipsilateral Condylar and Emissary Veins Visualization Quality Without vs. With Balloon Inflation (N=17)

| **Assessment Category** | **Without Balloon** | **With Balloon** | **Absolute Difference** | **P-value** |
| --- | --- | --- | --- | --- |
| **Homogenous Contrast Opacification** |  |  |  |  |
| Both condylar and other emissary veins (ipsilateral) | 3.3 (19.6%) | 11.0 (64.7%) | +45.1% | <0.001*** |
| Condylar veins only (ipsilateral) | 5.3 (31.4%) | 2.3 (13.7%) | -17.6% | 0.035* |
| Homogenous opacification not observed anywhere | 4.0 (23.5%) | 1.3 (7.8%) | -15.7% | 0.077 |
| Other emissary veins only (ipsilateral) | 4.3 (25.5%) | 2.3 (13.7%) | -11.8% | 0.238 |
| **Clear Vein Margins** |  |  |  |  |
| Both condylar and other emissary veins (ipsilateral) | 3.7 (21.6%) | 11.0 (64.7%) | +43.1% | <0.001*** |
| Condylar veins only (ipsilateral) | 5.3 (31.4%) | 2.3 (13.7%) | -17.6% | 0.035* |
| Not clear at all | 3.3 (19.6%) | 1.3 (7.8%) | -11.8% | 0.180 |
| Other emissary veins only (ipsilateral) | 4.7 (27.5%) | 2.3 (13.7%) | -13.7% | 0.143 |
| **Furthest Contrast Visualization** |  |  |  |  |
| Both condylar and other emissary veins (ipsilateral) | 4.3 (25.5%) | 11.7 (68.6%) | +43.1% | <0.001*** |
| Condylar veins only (ipsilateral) | 6.3 (37.3%) | 2.0 (11.8%) | -25.5% | <0.001*** |
| Not applicable | 1.7 (9.8%) | 1.0 (5.9%) | -3.9% | 0.727 |
| Other emissary veins only (ipsilateral) | 4.7 (27.5%) | 2.3 (13.7%) | -13.7% | 0.092 |
| **Ipsilateral Cortical Veins Visualization** |  |  |  |  |
| Visible | 1.0 (5.9%) | 11.0 (64.7%) | +58.8% | <0.001*** |
| Not visible | 16.0 (94.1%) | 6.0 (35.3%) | -58.8% | <0.001*** |

**Note:** Patient counts represent averages across three reviewers. *p<0.05, **p<0.01, ***p<0.001.

**Table S9.** Ipsilateral Condylar and Emissary Veins Visualization Quality by Individual Reviewer (N=17)

| **Assessment Category** | **Reviewer #1** | **Reviewer #2** | **Reviewer #3** | **Average** |
| --- | --- | --- | --- | --- |
| **WITHOUT BALLOON INFLATION** |  |  |  |  |
| **Homogenous Contrast Opacification, n (%)** |  |  |  |  |
| Both condylar and other emissary veins (ipsilateral) | 1 (5.9%) | 4 (23.5%) | 5 (29.4%) | 3.33 (19.6%) |
| Condylar veins only (ipsilateral) | 12 (70.6%) | 2 (11.8%) | 2 (11.8%) | 5.33 (31.4%) |
| Homogenous opacification not observed anywhere | 4 (23.5%) | 4 (23.5%) | 4 (23.5%) | 4.00 (23.5%) |
| Other emissary veins only (ipsilateral) | 0 (0.0%) | 7 (41.2%) | 6 (35.3%) | 4.33 (25.5%) |
| **Clear Vein Margins, n (%)** |  |  |  |  |
| Both condylar and other emissary veins (ipsilateral) | 1 (5.9%) | 5 (29.4%) | 5 (29.4%) | 3.67 (21.6%) |
| Condylar veins only (ipsilateral) | 12 (70.6%) | 2 (11.8%) | 2 (11.8%) | 5.33 (31.4%) |
| Not clear at all | 4 (23.5%) | 2 (11.8%) | 4 (23.5%) | 3.33 (19.6%) |
| Other emissary veins only (ipsilateral) | 0 (0.0%) | 8 (47.1%) | 6 (35.3%) | 4.67 (27.5%) |
| **Furthest Contrast Visualization, n (%)** |  |  |  |  |
| Both condylar and other emissary veins (ipsilateral) | 1 (5.9%) | 4 (23.5%) | 8 (47.1%) | 4.33 (25.5%) |
| Condylar veins only (ipsilateral) | 15 (88.2%) | 2 (11.8%) | 2 (11.8%) | 6.33 (37.3%) |
| Not applicable | 1 (5.9%) | 3 (17.6%) | 1 (5.9%) | 1.67 (9.8%) |
| Other emissary veins only (ipsilateral) | 0 (0.0%) | 8 (47.1%) | 6 (35.3%) | 4.67 (27.5%) |
| **Ipsilateral Cortical Veins Visualization, n (%)** |  |  |  |  |
| Visible | 2 (11.8%) | 0 (0.0%) | 1 (5.9%) | 1.00 (5.9%) |
| Not visible | 15 (88.2%) | 17 (100.0%) | 16 (94.1%) | 16.00 (94.1%) |
|  |  |  |  |  |
| **WITH BALLOON INFLATION** |  |  |  |  |
| **Homogenous Contrast Opacification, n (%)** |  |  |  |  |
| Both condylar and other emissary veins (ipsilateral) | 9 (52.9%) | 11 (64.7%) | 13 (76.5%) | 11.00 (64.7%) |
| Condylar veins only (ipsilateral) | 7 (41.2%) | 0 (0.0%) | 0 (0.0%) | 2.33 (13.7%) |
| Homogenous opacification not observed anywhere | 1 (5.9%) | 2 (11.8%) | 1 (5.9%) | 1.33 (7.8%) |
| Other emissary veins only (ipsilateral) | 0 (0.0%) | 4 (23.5%) | 3 (17.6%) | 2.33 (13.7%) |
| **Clear Vein Margins, n (%)** |  |  |  |  |
| Both condylar and other emissary veins (ipsilateral) | 9 (52.9%) | 11 (64.7%) | 13 (76.5%) | 11.00 (64.7%) |
| Condylar veins only (ipsilateral) | 7 (41.2%) | 0 (0.0%) | 0 (0.0%) | 2.33 (13.7%) |
| Not clear at all | 1 (5.9%) | 2 (11.8%) | 1 (5.9%) | 1.33 (7.8%) |
| Other emissary veins only (ipsilateral) | 0 (0.0%) | 4 (23.5%) | 3 (17.6%) | 2.33 (13.7%) |
| **Furthest Contrast Visualization, n (%)** |  |  |  |  |
| Both condylar and other emissary veins (ipsilateral) | 11 (64.7%) | 11 (64.7%) | 13 (76.5%) | 11.67 (68.6%) |
| Condylar veins only (ipsilateral) | 6 (35.3%) | 0 (0.0%) | 0 (0.0%) | 2.00 (11.8%) |
| Not applicable | 0 (0.0%) | 3 (17.6%) | 0 (0.0%) | 1.00 (5.9%) |
| Other emissary veins only (ipsilateral) | 0 (0.0%) | 3 (17.6%) | 4 (23.5%) | 2.33 (13.7%) |
| **Ipsilateral Cortical Veins Visualization, n (%)** |  |  |  |  |
| Visible | 11 (64.7%) | 13 (76.5%) | 9 (52.9%) | 11.00 (64.7%) |
| Not visible | 6 (35.3%) | 4 (23.5%) | 8 (47.1%) | 6.00 (35.3%) |

**REDCap Survey**

Which side's venous system is dominant?	Right side Left side Codominant

Not able to assess

Mean diameter of the internal jugular vein (mm), as measured on DSA at the C1 transverse process.

Which side was the balloon placed on?	Right Left

Location of balloon	Transverse sinus

Sigmoid sinus Internal jugular vein

Size of balloon (maximum inflated diameter in mm)

Any contrast seen filling downstream of the inflated balloon?	Yes No

What are all the structures visible upstream of the Ipsilateral sigmoid sinus

IJV without balloon inflation? Ipsilateral transverse-sigmoid junction

 Ipsilateral transverse sinus

- Ipsilateral superior petrosal sinus
- Ipsilateral inferior petrosal sinus
- Ipsilateral cavernous sinus

Ipsilateral bridging veins

 Ipsilateral cortical veins

Ipsilateral deep venous structures

 Torcula

 Superior sagittal sinus Straight sinus

 Inferior sagittal sinus

 Contralateral sigmoid sinus

Contralateral transverse-sigmoid junction

 Contralateral transverse sinus

Contralateral superior petrosal sinus

Contralateral inferior petrosal sinus

 Contralateral cavernous sinus

Contralateral bridging veins

 Contralateral cortical veins

Contralateral deep venous structures

 Not applicable

What are all the structures visible upstream of the Ipsilateral sigmoid sinus

IJV with balloon inflation? Ipsilateral transverse-sigmoid junction

 Ipsilateral transverse sinus

Ipsilateral superior petrosal sinus

Ipsilateral inferior petrosal sinus

 Ipsilateral cavernous sinus

Ipsilateral bridging veins

 Ipsilateral cortical veins

Ipsilateral deep venous structures

 Torcula

 Superior sagittal sinus Straight sinus

 Inferior sagittal sinus

 Contralateral sigmoid sinus

Contralateral transverse-sigmoid junction

 Contralateral transverse sinus

Contralateral superior petrosal sinus

Contralateral inferior petrosal sinus

 Contralateral cavernous sinus

Contralateral bridging veins

 Contralateral cortical veins

Contralateral deep venous structures

 Not applicable

What is the most distant landmark, upstream of the	Ipsilateral sigmoid sinus

IJV, where you can visualize contrast filling without	Ipsilateral transverse-sigmoid junction balloon inflation?	Ipsilateral transverse sinus

Superior sagittal sinus

Contralateral transverse sinus

Contralateral transverse-sigmoid junction

Contralateral sigmoid sinus

Not applicable

What is the most distant landmark, upstream of the	Ipsilateral sigmoid sinus

IJV, where you can visualize contrast filling with	Ipsilateral transverse-sigmoid junction balloon inflation?	Ipsilateral transverse sinus

Superior sagittal sinus

Contralateral transverse sinus

Contralateral transverse-sigmoid junction

Contralateral sigmoid sinus

Not applicable

**Visualization of the ipsilateral lateral skull base sinus without balloon inflation**

Is there homogenous contrast opacification?	Sigmoid sinus only

Up till the transverse-sigmoid junction Both transverse and sigmoid sinuses

Homogenous opacification not observed anywhere

Are margins of the lateral skull base sinus clear?	Ipsilateral sigmoid sinus only

Up till the ipsilateral transverse-sigmoid junction

Both ipsilateral transverse and sigmoid sinuses

Not clear at all

What is the furthest you can visualize contrast in the	Ipsilateral sigmoid sinus only

sinus?	Up till the ipsilateral transverse-sigmoid junction

Both ipsilateral transverse and sigmoid sinuses

Torcula or beyond

Not applicable

**Visualization of the ipsilateral lateral skull base sinus with balloon inflation**

Is there homogenous contrast opacification?	Sigmoid sinus only

Up till the transverse-sigmoid junction Both transverse and sigmoid sinuses

Homogenous opacification not observed anywhere

Are margins of the lateral skull base sinus clear?	Ipsilateral sigmoid sinus only

Up till the ipsilateral transverse-sigmoid junction

Both ipsilateral transverse and sigmoid sinuses

Not clear at all

What is the furthest you can visualize contrast in the	Ipsilateral sigmoid sinus only

sinus?	Up till the ipsilateral transverse-sigmoid junction

Both ipsilateral transverse and sigmoid sinuses

Torcula or beyond

Not applicable

**Visualization of the condylar and other emissary veins (ipsilateral) without balloon inflation**

Is there homogenous contrast opacification?	Condylar veins only (ipsilateral)

Other emissary veins only (ipsilateral)

Both condylar and other emissary veins (ipsilateral)

 Homogenous opacification not observed anywhere

Are margins of the condylar and other emissary veins	Condylar veins only (ipsilateral)

clear?	Other emissary veins only (ipsilateral)

Both condylar and other emissary veins (ipsilateral)

Not clear at all

What is the furthest you can visualize contrast in these?	Condylar veins only (ipsilateral)	Other emissary veins only (ipsilateral)

Both condylar and other emissary veins (ipsilateral)

Not applicable

**Visualization of the condylar and other emissary veins (ipsilateral) with balloon inflation**

Is there homogenous contrast opacification?	Condylar veins only (ipsilateral)

Other emissary veins only (ipsilateral) Both condylar and other emissary veins (ipsilateral)

Homogenous opacification not observed anywhere

Are margins of the condylar and other emissary veins clear?	Condylar veins only (ipsilateral)	Other emissary veins only (ipsilateral)

Both condylar and other emissary veins (ipsilateral)

Not clear at all

What is the furthest you can visualize contrast in these?	Condylar veins only (ipsilateral)	Other emissary veins only (ipsilateral)

Both condylar and other emissary veins (ipsilateral)

Not applicable

**Visualization of ipsilateral cortical veins**

Do you visualize any ipsilateral cortical veins on	Yes

retrograde injection without balloon inflation?	No

Do you visualize any ipsilateral cortical veins on	Yes

retrograde injection with balloon inflation?	No

**Visualization of pathology**

How well can you visualize the pathology without balloon inflation?

Well

Somewhat well

Not very well

How well can you visualize pathology with balloon inflation?

Well

Somewhat well

- Not very well
